# Non-invasive visualization of physiological changes of insects during metamorphosis based on biophoton emission imaging

**DOI:** 10.1038/s41598-019-45007-3

**Published:** 2019-06-12

**Authors:** Shoko Usui, Mika Tada, Masaki Kobayashi

**Affiliations:** 1 Graduate Department of Electronics, Tohoku Institute of Technology, Sendai, 982-8577 Japan; 2 Center for General Education, Tohoku Institute of Technology, Sendai, 982-8577 Japan

**Keywords:** Entomology, Applied optics

## Abstract

Spontaneous ultra-weak photon emission from living organisms, designated as biophoton emission, is a generally observed phenomenon irrespective of the organism species. Biophoton emission is attributed to the production of excited molecules in a metabolic biochemical reaction, especially in processes involving reactive oxygen species (ROS). Although many plant and mammal subjects have reportedly been used to study its application to biological measurements, biophoton emission properties of insects remain unclear. For this study, we strove to measure the variation of two-dimensional images of biophoton emission during the metamorphosis of lepidopterous insects as a moving picture to elucidate the physiological changes underlying the mechanism of drastic changes of morphological and ecological characteristics of the insects. We used our developed biophoton imaging system incorporating a cooled charge-coupled device (CCD) camera and a specially designed lens system to elucidate the spatiotemporal dynamics of biophoton emission during metamorphosis, larval–pupal ecdysis/pupation of *Papilio protenor*, suggesting its applicability for *in vivo* observation of physiological changes during the regulation of metamorphosis.

## Introduction

Ultra-weak photons emerged from living organisms are often designated as biophoton emission^1^, originating from chemically excited molecules produced in cellular biochemical reactions in various metabolic processes without photo-excitation^1–5^. This spontaneous photon emission is distinguished from generally known bioluminescence, which is visible light emission based on the luciferin–luciferase mechanism from organisms of specific species. Biophoton emission is attributed to the constituents of living materials such as lipids, protein, and DNA. Through their interaction with reactive oxygen species (ROS) and/or free radicals, excited species are produced by decomposition of high-energy intermediates derived in enzymatic or non-enzymatic reaction processes^3–8^. For example, in the state of normal metabolism or the state induced by some stress, mitochondrial electron transfer chain is known to be a ROS source leading to biophoton emission. The biophoton emission intensity is generally less than 10^−16^ W/cm^2^, which is 3–6 order lower than the light intensity that is visible to the naked eye, but the wavelengths of emissions normally extend over the entire visible wavelength. For the imaging of biophoton emissions, a highly sensitive imaging apparatus with sufficient sensitivity to detect low levels of light such as the state of a single photoelectron event are required^9^. In the case of plants, biophoton emission from germinating roots^10^ under the normal metabolic conditions or various environmental stress^10–13^ have been reported. Virus-infected leaves also exhibit remarkable photon emission that is synchronized with the emergence of a resistive response of the plant, known as hypersensitive response, originating from ROS secretion against virus proliferation^14,15^. In mammals, biophoton emissions reportedly reflect the metabolic activity of rat brain^16^. Disease-specific biophoton emission has been observed in other animals. Biophoton imaging of cancer-transplanted mice^17,18^ and mice with artificially induced rheumatoid arthritis^19,20^ were demonstrated by our team while pursuing an application to evaluate oxidative stress and disease. Human beings also exhibit biophoton emission^21–23^ reflecting metabolic activity, which is synchronized with the circadian rhythm^22^, a state of oxidative stress of the skin affected by ultraviolet (UV) light^24–27^ or other environmental factors. Results of these numerous studies suggest the potential application of biophoton detection for non-invasive measurement of physiological and pathological properties of living bodies. Especially, imaging^9^ and spectroscopy^23^ techniques of biophoton emission are crucially important for exploring its applications and associated mechanisms.

Biophoton studies of insects that are not specifically bioluminescent species have been less reported. Particularly, no report of the relevant literature describes imaging of biophoton emissions. To pursue non-invasive measurement of biological information of insects, we have strived to collect two-dimensional images and their temporal variation during insect metamorphosis. Actually, insect metamorphosis is a mystic and spectacular event performed in an extremely short period, yet achieving dramatic changes of morphological and physiological characteristics. The physiological mechanisms for orchestration of its complex processes fascinate us and arouse our curiosity. The hormonal mechanism with signaling pathway and corresponding transcription factors conducting metamorphosis are explored in detail. It is interpreted to initiate by the coordination of some hormones represented by molting hormone ecdysteroids^28–30^. Ecdysteroid hormones are synthesized and secreted in prothoracic glands (PG), playing crucially important roles in the regulation of metamorphosis commonly in widely diverse insects. Synthesis of ecdysteroids in the PG is stimulated primarily by a neuropeptide prothoracicotropic hormone (PTTH) secreted by brain neurosecretory cells, temporally governing molting processes and metamorphosis. After receiving a signal on the receptor of PTTH in PG, the complex network of signal transduction activates ecdysteroidogenesis with the cooperation of various signal mediators. In the PG, cholesterol is converted into ecdysone through a series of ecdysteroidogenic enzymes. Secreted ecdysone into hemolymph is transformed to steroid hormone 20-hydroxyecdysone (20E), which initiates gene expression cascade responsible for molting processes. In larval–pupal metamorphosis/pupation, an early step of this process induces apolysis: the detachment of epidermal cell layers from old cuticles and synthesis of a new cuticle layer^30^. This stage before the pupal ecdysis, called the “pharate” stage of the pupa or pre-pupa, involves the remodeling of larval tissue through programmed cell death (PCD), which regulates the histolysis of obsolete larval tissues and larval fat body transformation by autophagy and apoptosis^30–32^.

As described above, biophoton emission originates in high-energy products derived from enzymatic or non-enzymatic processes of metabolism, chiefly associated with the chemical reaction involving ROS and free radicals. In fact, ROS-induced oxidative damage to cellular constituents is a typical mechanism implicated in biophoton emission. Traditionally, ROS are viewed as undesirable by-products generated in oxidative metabolism processes, causing oxidative stress, and subsequently leading to disease and aging. Recently however, the physiological role of ROS serving as signaling molecules for the regulation of physiological function is attracting attention^33^. During metamorphosis, ROS-mediated signaling and their crucially important roles are coming to be identified^34,35^. The view of redox biology is arising, in which a small increase of mitochondria-derived ROS is interpreted to activate a variety of initial processes of signal transduction. The production of ROS through both sides of the properties is possibly connected to biophoton emission.

As described herein, we present the first report based on observed biophoton images evolved in the process of metamorphosis of lepidopteran insects, visualizing the dynamics of physiological properties as a moving picture of biophoton emission.

## Results

**Figure 1:**
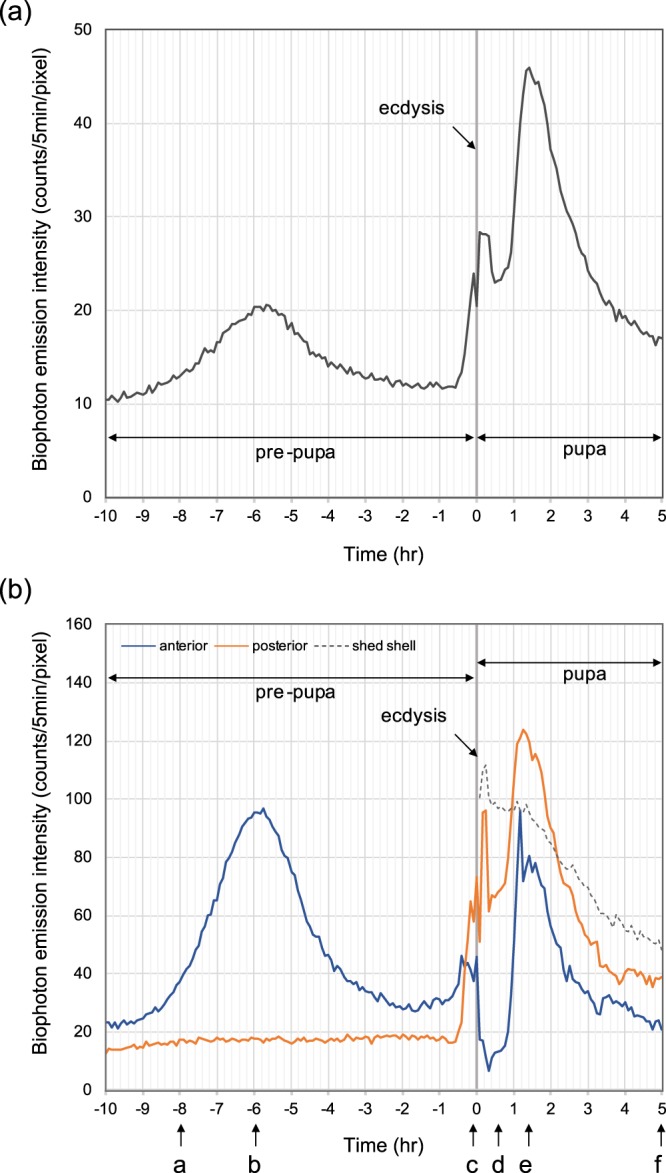
Time course of biophoton emission intensity of *Papilio protenor* from 10 hr before pupal ecdysis to 5 hr after ecdysis. (**a**) Intensity time course of the whole body of the subject. (**b**) Intensity time course from the anterior region (blue line) and posterior region (orange line). After ecdysis, photon emission from a shed-shell is also shown (dotted line). Characters a–f with arrows denote the time points of biophoton images displayed in Fig. 2.

Figure 1 presents the time course of biophoton emission intensity during pupation from the pre-pupal stage to the pupal stage of *Papilio protenor*, including pupal ecdysis (molting). The timescale in the figures is presented with the molting timing at 0 hr, designating before (−) and after (+) the ecdysis. The figures show results obtained from 10 hr before ecdysis to 5 hr after ecdysis. At time 0, ecdysis behavior accompanied by intensive body movement was completed within 5 min (integration time for a single image acquisition). The time course of the intensity obtained from the whole body of the insect, displayed in Fig. 1(a), is characterized by two major peaks of photon emissions appearing at 6 hr before ecdysis and 1.5 hr after ecdysis. Small peaks with short duration were also distinguished directly before and after ecdysis. Figure 1(b) shows the time course of biophoton emission from two regions of interest (ROIs) fixed at the anterior part and the posterior part of the insect, representing the position-specific biophoton emission. Augmentation of biophoton emission during the pre-pupal stage was anterior-specific, in which the intensity increased from 9 hr before ecdysis, and reached a maximum at 3 hr (6 hr before ecdysis); then it decreased to the original level within 4 hr (approx. 2 hr before ecdysis). This temporal augmentation of the intensity was localized at the anterior region. By contrast, photon emission intensity in the posterior part was flat. Not even a small change was observed in this period. At 40 min before ecdysis, the photon emission intensity in the anterior part increased again rapidly. Simultaneously, a remarkable increase of photon emission from the posterior part was observed.

Right after ecdysis, photon emission from the anterior region sharply decreased to half the level of the intensity before ecdysis. After this darkened period of the anterior part lasting for 45 min, the intensity increased drastically and reached maximum intensity within 15 min. Subsequently, it decreased gradually to the original level within 2 hr. By contrast, the posterior region showed pulse-like emission with 10 min duration, but the intensity did not decline as in the anterior part. At 45 min after ecdysis, the intensity increased again. It proceeded with the increase of anterior region of approx. 10 min and peaked within 30 min, being coincident with the peak time of anterior region (75 min after ecdysis). Subsequently, it decreased gradually to a steady level during 2 hr. A distinctive pattern of spatiotemporal properties of biophoton emission was observed in this period from 10 hr before to 5 hr after ecdysis. To confirm reproducibility, we conducted multiple measurements using four subjects in the same condition. All subjects showed similar patterns of the time course of biophoton emission, presenting two major peaks before and after ecdysis. For the primal peak before ecdysis, the time difference among all subjects was ranged within 1 hr. Furthermore, for the major peak after ecdysis, the time difference was within 30 min (Comparison of the time course among all four measurements is provided as Supplementary Fig. S1).

**Figure 2:**
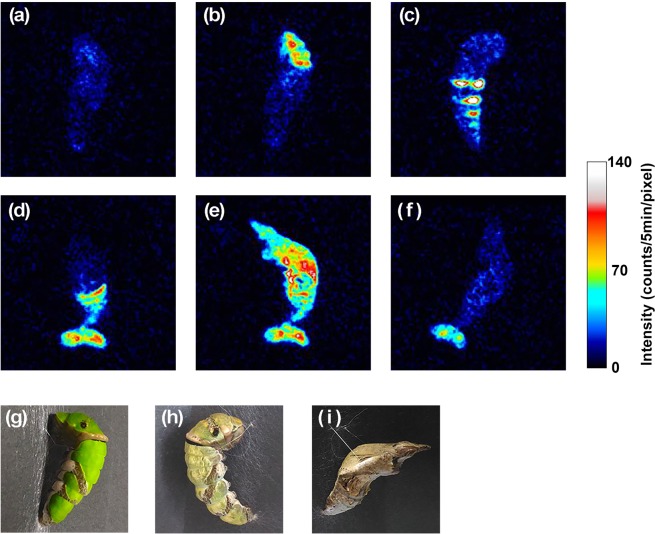
Biophoton images of *Papilio protenor* obtained at specific time points: (**a**) 8 hr before ecdysis, (**b**) 6 hr before ecdysis, (**c**) 5–10 min before ecdysis, (**d**) 20–25 min after ecdysis, (**e**) 75–80 min after ecdysis, and (**f**) 5 hr after ecdysis. (**g**) Picture of the subject in the larval stage taken before biophoton measurement. (**h**) Picture of the pre-pupa taken directly before ecdysis (different subject, not used for biophoton imaging). (**i**) Picture of the subject in the pupal stage taken after biophoton measurement. Time points of (**a**)–(**f**) are designated in Fig. 1(b) with corresponding characters and arrows.

Figure 2 shows six characteristic images at different time points during metamorphosis. The biophoton image of the pre-pupa (pharate stage) observed at 8 hr before pupation is displayed in Fig. 2(a), representing weak and uniform intensity of biophoton emission on the whole surface of the insect. A picture of the subject taken before biophoton measurement is also presented in Fig. 2(g). Figure 2(b) is an image taken at 6 hr before ecdysis, which corresponds to the major peak of biophoton emission before pupation, as suggested in the time course analysis displayed in Fig. 1(b). Photon emission was localized specifically in the anterior region with a few scattered spots. Figure 2(c) portrays an image taken at the time point directly before ecdysis: a remarkable increase of photon emission from the portion corresponding to abdominal segments was observed. The photon emission pattern observed at abdominal segments implies the onset of the early step of behavioral sequence of ecdysis, characterized as pre-ecdysis behavior of the movement for preparation of ecdysis. An image of a pre-pupa directly before pupal ecdysis, taken of a different subject for reference, is also presented in Fig. 2(h). Biophoton images observed after ecdysis are presented in Fig. 2(d)–(f). Figure 2(d) is an image obtained at 20–25 min after ecdysis, showing higher intensity of photon emission at the posterior side of the pupa and the emission from the shed shell attached on the tail. Photon emission from the shed shell decreased gradually during 5 hr after ecdysis, as represented in Fig. 1(b) by a dotted line. In contrast, photon emission from the posterior side increased and spread toward the anterior side of the body. It culminated at 75 min after ecdysis. Figure 2(e) is an image of biophoton emission at the maximum intensity (75–80 min after ecdysis), depicting biophoton emission from the whole body with the structural pattern, which seems to represent the pattern of a newly developed pupal cuticle. At 5 hr after ecdysis, photon emission decreased to the equivalent level of the pre-pupal stage, as presented in Fig. 2(f). A photograph of the pupa taken after the end of the measurement is attached in Fig. 2(i). A moving picture edited combining 181 biophoton images acquired during 15 hr from pre-pupa to pupa including the ecdysis for pupation is provided as a video in Supplementary Information (Supplementary Video S1). In the video, spatiotemporal changes of biophoton emission during the metamorphosis are visualized with comparison to the visual appearance of the insect, showing morphological changes taken using the different subject. Another video comparing the moving pictures of biophoton emission observed in four independent measurements using different subjects is provided to express the reproducibility (Supplementary Video S2).

## Discussion

Results revealed the characteristic variation of biophoton emission of insect during metamorphosis, representing spatiotemporal dynamics of physiological changes that emerged during the molting process. Biophoton emission is originated in the electronically excited molecules produced in various metabolic biochemical reactions. The process of metamorphosis is accompanied by drastic physiological changes, in which consecutive events conducting the transition of morphological and ecological properties are regulated precisely by the complex mechanism of signal transduction and gene expression. The behavior of biophoton emissions observed in this examination is suggested to reflect the notable events promoted in the sequence of metamorphosis. The first major peak of biophoton emission before ecdysis is localized in the anterior head region, implying the occurrence of the event specifically evolved at the anterior region in the molting process of the pharate stage of the pre-pupa. In this stage, an insect is encased in its old larval cuticle, but apolysis proceeds with the accompanying PCD process, of which an epidermal cell layer from the old cuticle detaches and molting enzymatic fluids secrete to dissolve the old cuticle for re-cycling^30,36^. In *Manduca sexta*, the resorption of molting fluid and appearance of air to inflate the new tracheal tubes is known to occur in the old head capsule at approx. 6 hr before pupal ecdysis^30^, which corresponds to the first major peak of biophoton emission. The source and mechanism of temporal augmentation of biophoton emission in the pharate stage is not understood clearly, but considering the anterior localized photon emission, it might originate in oxidative reaction of the molting fluid filled in the head capsule. Molting fluid, containing a high concentration of ROS^36^, also has the function of insect immunity: it protects the insect from infection during ecdysis^36^. The contribution of ROS that derives oxidization in the molting fluid is suspected to be involved in biophoton emission.

At approx. 1 hr before ecdysis, the pharate pupa launches behavioral sequence of molting^30,37,38^, being designated as pre-ecdysis I initiated by pre-ecdysis triggering hormone (PETH). Furthermore, it follows pre-ecdysis II induced by ecdysis triggering hormones (ETH); then it engenders ecdysis behavior. Biophoton emissions were observed on the whole body during this time, in which the characteristic augmentation at abdominal segments was recognized clearly. It apparently represents the inception of the pre-ecdysis behavioral sequence. During ecdysis behavior, biophoton emission decreases temporally. Subsequently, after finishing the molting, photon emission from the abdomen region increased again. It is particularly interesting that intensity from the anterior region was depressed, becoming weaker than that before ecdysis. After ecdysis, photon emission at the posterior region spread to the anterior region. It culminated within 1.5 hr after ecdysis. As a post-ecdysis process, tanning of the new pupal cuticle progresses. It is characterized by cuticle sclerotization and melanization with epidermis ommochrome synthesis triggered by a neuropeptide bursicon^30,39–41^. In *Bombyx mori*, activity of laccase, which is the phenoloxidase mediating the metabolic process of cuticle tanning, reportedly reaches a peak within 1–5 hr after ecdysis^42^. In the cuticle sclerotization and melanization process, free radical reaction^41^ and ROS generation^43^ are known to be involved, suggesting that a radical reaction triggered by ROS in this process can induce the excited state of melanin or other pigments leading to biophoton emission. During this post-ecdysis process, immediately after molting, the shed-shell touched at the tail also showed higher intensity of photon emission, which is stronger than the body of the molted pupa. Intense photon emission from the freshly shed shell gradually declined to the original level within 5 hr, suggesting that photon emission originated from oxidation of unsclerotized old cuticle with molting fluid in the shell. However, during larval–pupal metamorphosis, the PCD mechanism induces histolysis of obsolete larval tissues and their remodeling. In this process, mitochondria-derived ROS are known to be involved in the signaling^33^, which might be implicated in the generation of biophoton emissions.

Meanwhile, optical transmittance of the insect tissue in larvae or surface color changes during cuticle formation in pupae might affect the intensity of observed photon emissions. Visual observations during these processes revealed no apparent change of surface color along with the formation of emission peaks during ecdysis, suggesting that the intensity variations are attributable predominantly to changes in biophoton emissions.

In conclusion, this study revealed the spatiotemporal dynamics of biophoton emission during insect metamorphosis. Biophoton emission with its spatiotemporal distribution appears to provide raw information connected to physiological and ecological features underlying the driving mechanism of metamorphosis. However, detailed mechanisms leading to biophoton emission are still not determinate. Further study is necessary to elucidate the pathway and sources of biophoton emission observed as peaks formed in pre-pupal and pupal development.

Biophoton imaging technique might open new insights into measurement of the nature of insects. Knowledge in this area is expected to be helpful in investigating insect physiology for exploring agricultural applications such as pest control. Various species, such as fireflies, that have bioluminescent competence exist in the insect kingdom. Although biophoton emission is a general phenomenon appearing in all living organisms without bioluminescent competence, it implies that the properties of specific biophoton emission accompanied by physiological changes might give some clue to answering the question of how bioluminescent insects acquire luminous ability in the long-term history of biological evolution.

## Materials and Methods

### Animals

Lepidopteran swallowtail butterflies, the spangle *Papilio protenor* Cramer, 1775, were used as subjects. Eggs were taken from the field and were hatched at 23 °C. Larvae were reared on leaves of *Poncirus trifoliata* for 2 weeks after hatching. A five instar larva was reared on a sheet of black paper with a leaf. At 4 or 5 days after molting of 5 instar larvae, the insects transformed to pre-pupa stage and weaved a belt to hook the body on the black paper. The pre-pupa fixed on the black paper was used as a subject for biophoton measurement.

### Biophoton imaging system

The biophoton imaging system consists of a highly sensitive cooled charge-coupled-device (CCD) camera and a specially designed high-throughput lens system. The CCD camera (850 series; Spectral Instruments, Inc., AZ, USA) incorporates a back-illuminated CCD (CCD42-40; Teledyne e2v, UK) operated under −85 °C cooling. The effective area of the CCD is 27.6 × 27.6 mm with 2048 × 2048 pixel format. The CCD camera was operated in 8 × 8 binning mode, resulting in the actual pixel number of 256 × 256. The specially designed lens system, which is the same as that used for biophoton imaging of mice^17,18^, has high throughput performance with a 0.5 numerical aperture (NA) from the image plane side. The lens magnification is 1/3, corresponding to the imaging area of 75 × 75 mm with 300 μm spatial resolution. Observable wavelengths of the system are 400–850 nm. The minimum detectable photon number emitted on the subject surface was estimated as 330 photon/s/cm^2^, corresponding to 1.1 × 10^−16^ W/cm^2^ in each binned pixel under the condition of 5 min integration time and assuming 600 nm wavelength (described in detail in Supplementary Methods).

The subject was installed in a light-tight dark box placed in a dark room. The lens system was equipped in the dark box. The CCD camera was attached with the lens system through the top plate of the dark box. The focusing operation was done with height control of the sample stage by a translational auto-stage incorporated in the dark box (a schematic of the system is portrayed in Supplementary Fig. S2).

### Measurement procedure

The subject of pre-pupa fixed on the black paper was put on a black plastic dish (70 mm diameter) and was installed in the dark box with ambient temperature of 25 °C. The biophoton image was measured continuously for 24 hr covering the pupal metamorphosis from larval pre-pupa to pupa. The CCD camera was operated with the exposure (integration) time of 5 min in each; 300 consecutive images were acquired. The measurement experiment was repeated with four independent subjects under the same conditions.

### Data processing

For the temporal analysis of biophoton intensity, the average count of the intensity accumulated in a ROI, which was set with an inscribed quadrangle including the insect, was extracted from all image data. The ROI was divided further to two ROIs of an anterior part and a posterior part of the insect. The anterior region was designated as including the head region of the larva and pupa. The divided ratio of both areas was approximately 1:2 for anterior and posterior region (see Supplementary Fig. S3). Noise reduction to eliminate spot-like spurious events caused by high-energy particles such as cosmic rays was conducted using 3 × 3 median filter before ROI analysis. All images presented in figures were depicted with pseudo color.

## Supplementary information

Supplementary data of the article: “Non-invasive visualization of physiological changes of insects during metamorphosis based on biophoton emission imaging”

Supplementary Video S1

Supplementary Video S2

## Data Availability

The raw data are available on request from corresponding authors.
