# Supplementary material for: Non-invasive visualization of physiological changes of insects during metamorphosis based on biophoton emission imaging
Source: Sci Rep. 2019 Jun 12;9:8576. doi: 10.1038/s41598-019-45007-3 (PMC6561905; doi:10.1038/s41598-019-45007-3)
Supplement: Supplementary file 1 — Supplementary data of the article: “Non-invasive visualization of physiological changes of insects during metamorphosis based on biophoton emission imaging” [file 41598_2019_45007_MOESM1_ESM.pdf]

Supplementary data of the article: "Non-invasive visualization of physiological changes of insects during metamorphosis based on biophoton emission imaging"

Shoko Usui [1], Mika Tada [2] & Masaki Kobayashi [1, *]

[1] Graduate Department of Electronics, Tohoku Institute of Technology, Sendai 982-8577, Japan

[2] Center for General Education, Tohoku Institute of Technology, Sendai 982-8577, Japan

* Correspondence and requests for materials should be addressed to Masaki Kobayashi (masaki@tohtech.ac.jp)

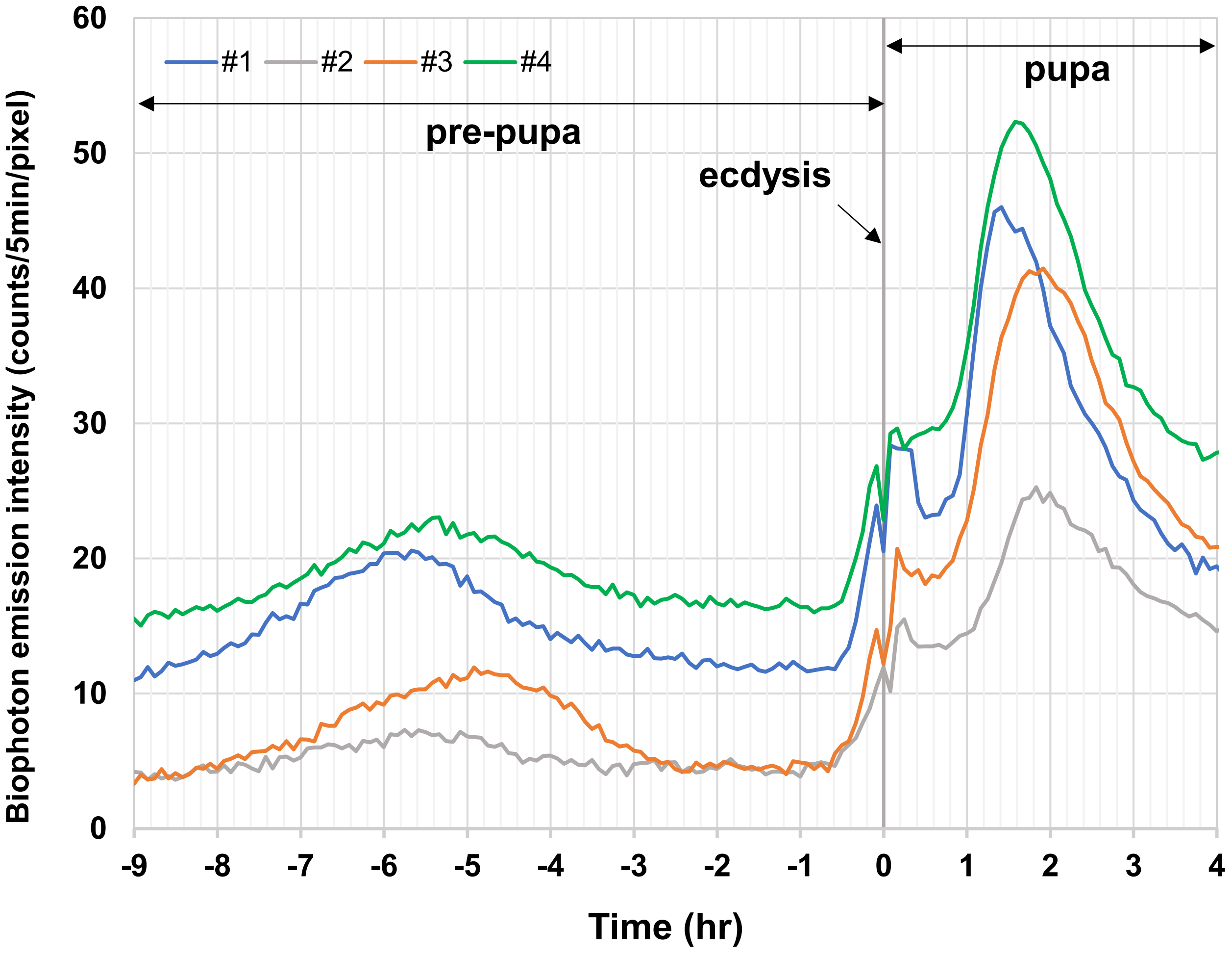

Supplementary Figure S1. Comparison of the time course of biophoton emission intensity observed from four independent subjects (#1 – #4) of *Papilio protenor.*

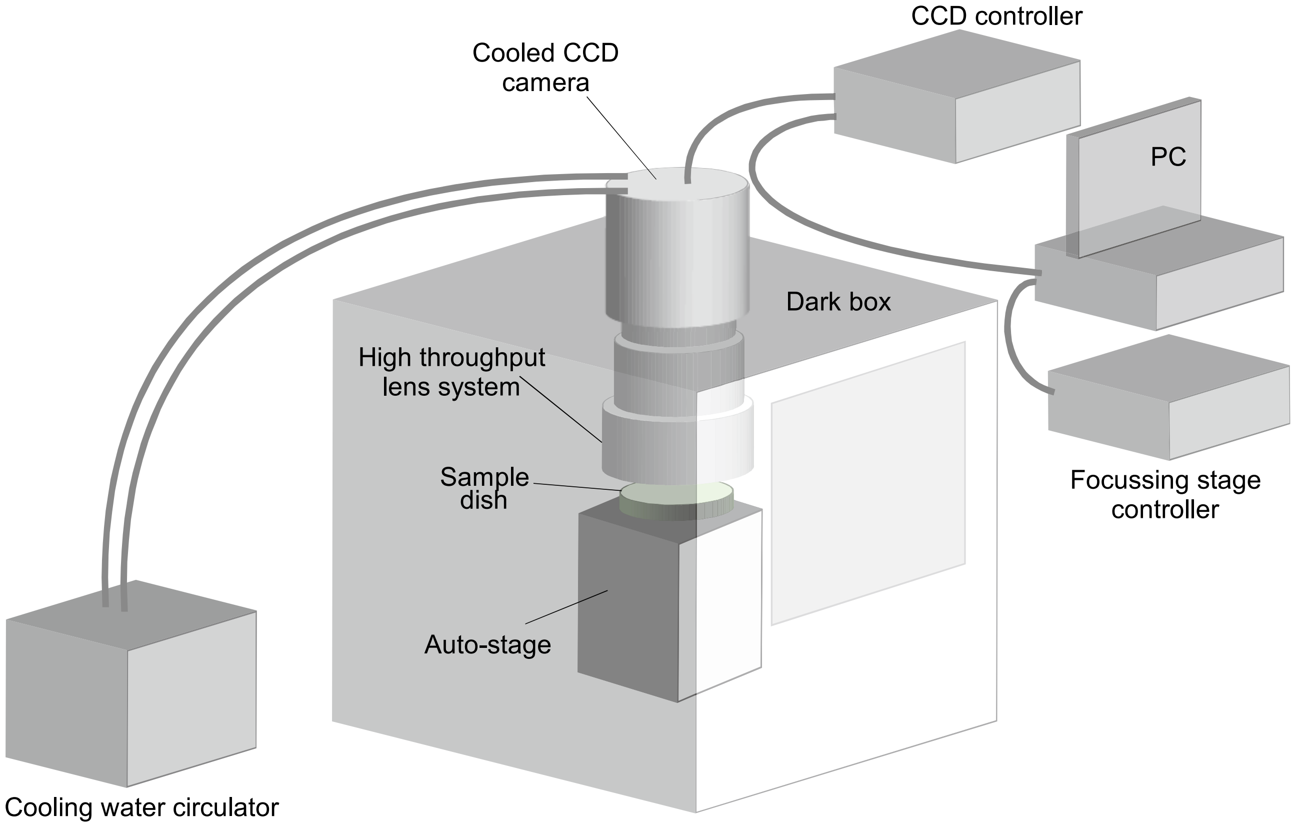

Supplementary Figure S2. Schematic of the experimental setup used for imaging of biophoton emission designed for the insect subjects.

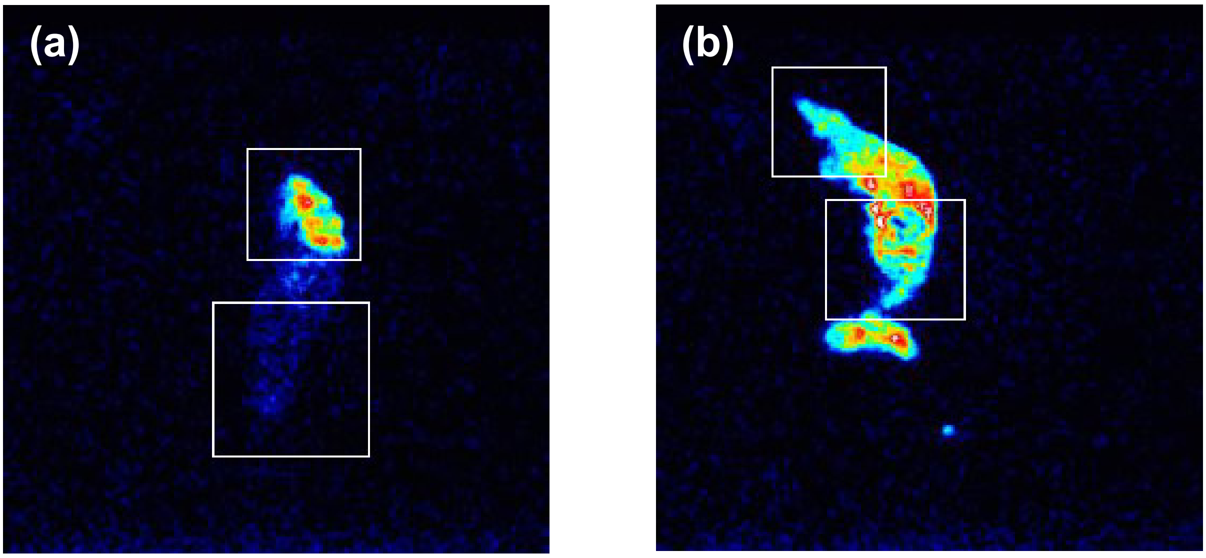

Supplementary Figure S3. Regions of interest (ROIs) for anterior and posterior region set for a pre-pupal larva (a) and a pupa (b) after ecdysis.

Supplementary Methods: Estimating the minimum detectable photon number of the system

The minimum detectable photon number can be derived from the signal-to-noise ratio (SNR) equation as

$$\frac{S}{N} = \frac{\eta\langle N_p\rangle T}{\sqrt{\langle N_d\rangle T + {N_r}^2}}$$

Here, $\langle N_p\rangle$ and $\langle N_d\rangle$ respectively represent the averaged photon number and averaged dark electron counts per pixel in unit time. N_r denotes the readout noise of output amplifiers expressed as the root-mean-square of the electron number. η and T respectively denote the quantum efficiency of the CCD and the exposure time for imaging. The minimum detectable photon number $\langle N_p\rangle_{min}$ is definable in the condition with $S/N = 1$. Therefore, it is derived as follows.

$$\langle N_p\rangle_{min} = \frac{\sqrt{\langle N_d\rangle T + {N_r}^2}}{\eta T}$$

The minimum detectable photon number emitted on the subject surface in unit area $\langle N_s\rangle_{min}$ is derived as shown below using magnification ratio M, the light collection efficiency of the lens system K, and pixel area S.

$$\langle N_s\rangle_{min} = \frac{\langle N_p\rangle_{min}\ M^2}{KS}$$

Parameter values of the system (in 8 × 8 binning mode) under the measurement condition are presented below.

$\langle N_d\rangle$=7.04×10^{-3} (e$^-$/s/pixel), N_r=2.91 (e$^-$ rms), η=0.92 (at wavelength 600 nm),
M=0.33, K=0.034, S=1.17 × 10^{-4} (cm^2), T=300 (s)

Consequently, $\langle N_s\rangle_{min}$= 330 (photon number/s/cm^2) is derived, which can be converted to optical power of 1.10×10^{-16} (W/cm^2) assuming 600 nm wavelength.

Supplementary Video S1. Moving image edited to combine all images of biophoton emission from 10 hr before pupal ecdysis to 5 hr after ecdysis (left video). For reference, a video showing the visual appearance of morphological changes taken using the different subject is attached (right video; starting from 5 hr before the ecdysis). An inserted graph (same as Fig. 1 (a)) indicates the timing of the video.

Supplementary Video S2. Moving images of biophoton emission compared among four independent subjects in the period from 9 hr before ecdysis to 4 hr after ecdysis.
